# Male partners and medical staff's perception on contributing factors of Family Planning (FP) in Fiji

**DOI:** 10.1016/j.heliyon.2021.e06068

**Published:** 2021-01-24

**Authors:** Mohammed Imtishal, Masoud Mohammadnezhad

**Affiliations:** School of Public Health and Primary Care, Fiji National University, Suva, Fiji Islands, Fiji

**Keywords:** Family Planning, Perception, Contributing factors, Male partners, Medical staff, Fiji

## Abstract

**Introduction:**

Male partners play an important role in Family Planning (FP) as they are directly involved in the decision making of FP with their partners. Medical staff are also involved with FP services that have a direct relation to usage of FP among the men and women. This study is aimed to explore the perception of male partners and the medical staff about factors affecting FP decision in Fiji.

**Materials and methods:**

The qualitative study was carried out among men who were partners of the pregnant women, and medical staff at Ba Mission Hospital (BMH), Fiji in 2019. The study utilised semi-structured questionnaire to guide four Focus Group Discussions (FGDs) among 10 male partners and 10 medical staff. Interview was audio transcribed and analyzed using thematic analysis.

**Result:**

The common themes identified from the male interviews were the opposition of male partners, religious norms and cultural beliefs, accessibility of contraception, and previous FP failure. The common themes identified from the medical staff interviews were; effectiveness of counseling, concerns on men involvement in FP, and barriers towards FP.

**Conclusion:**

This study reveals the need for better counseling, more awareness on FP for men and more involvement of men by the medical staff in FP practices.

## 1 Introduction

Family Planning (FP) is defined as “the conscious effort of couples and individuals to plan for and attain desired number of children and to regulate the spacing and timing of their births” (IPPF, 2011). Over 200 million women in resource constraint nations do not desire pregnancies and they fall short of using modern contraceptive methods and this contributes to the increasing fertility rates which have an impact on Maternal and Child Health (MCH) (Salisbury et al., 2016). Pacific Island Countries (PICs) have a Contraception Prevalence Rate (CPR) of less than 40% as compared to 66–85% for western countries (WHO Factsheet, 2018).

The practice of FP is constricted due to factors such as socio-cultural and psychological norms, lack of education and misinformation (Sharma et al., 2012) Also, fear of side effects, unavailability of services, and male partner's preferences are other compelling reasons limiting FP (Kamruzzaman and Hakim, 2015 and Schultz et al., 2018). The decision of family is affected by the male partner as women whose male partner refuses FP are 19% less likely to use contraception as compared to those where the male partners accept FP (Mboane and Bhatta, 2015). For some women their male partners do not allow them to practice FP for various reasons such as social stigma, family pressure, and disapproval from elders in the family who discourage FP use also (Gonie, 2018). On the other hand, some men are supportive of women and their decision of FP (Kofi et al., 2018). Previous studies conducted identified that there was lack of information about male contraception, the side effects of female contraception disrupted sexual life, some believed that contraception was only for females, while others shared their fear of extramarital affairs due to females using contraception and some disapproved as they wanted large families (Kabegenyi et al., 2012; Apanga and Adam, 2015; Tilahun et al., 2013; Schultz et al., 2018).

The importance of medical staff cannot be further emphasized as they play a vital role in FP by disseminating pertinent information pertaining to the women and ensure that they get the best advice possible (Kohan et al., 2014; Tshitenge et al., 2018). It was noted that the level of knowledge and attitude toward FP was relatively low and FP utilization was quite low among the healthcare workers (Wani et al., 2019). Therefore, it has to be seen whether the role that the medical staff play actually helps or that there role needs to be strengthened.

There is a dearth in studies on the perception of male partners and medical staff to FP in Fiji, hence it is exigent to conduct studies in this area in order to ascertain sufficient data focusing on male and medical staff involvement in FP. Thus, he aim for the study is to explore the perception of male partners and the medical staff about factors affecting FP decision in Ba Subdivision, Fiji.

## 2 Materials and methods

This qualitative study was conducted in Ba Mission Hospital (BMH), Fiji from 15^th^ April, 2019–15^th^ June, 2019. Focus Group Discussions (FGDs) were carried out among the partners and the medical staff using semi-structured questionnaires consisting of 9 open-ended questions for husbands and 7 open-ended questions for the medical staff which was developed by reviewing literature to answer the research questions. To make sure the questions are understandable and are in line with the research questions a pilot study was conducted among a small sample of male partners and medical staff. The questionnaires then were modified based on the comments provided by them.

The criteria for the male participants were all the male partners of the pregnant women, legally married or are in defacto relationship (live with each other but not legally married), attended the clinic with the pregnant women, self-identified Fijian, agreed to participate and of all religious and ethnic background. These study excluded male partners who were not interested in taking part in study or were in the clinic without their pregnant women. As the ideal number in each FGD is between 5-8 participants (Krueger and Mary, 2000), Ten participants in two FGDs (including 5 in each) who met the inclusion and exclusion criteria were selected using purposive sampling. Two FGDs was chosen based on the data saturation. The medical staff was selected using purposive sampling for two FGDs. This study included doctors, staff nurses, and registered midwives who are involved in ANC and Maternity Unit at Ba Mission Hospital, agreed to participate in the study and medical staff of all ethnic and religious background. This study excluded medical staff that did not look after the pregnant women and those unwilling to participate. The Maternity Ward had 10 medical staff including the doctors, nurses, and registered midwives who were regularly involved in FP. There were two FGDs at Ba Mission Hospital and since they did shift work, having 2 FGDs allowed them to participate. The sample size included a minimum of 1 doctor, 1 or 2 nurses and 2 or 3 midwife in each FGD.

The participants were informed about the study with Participant Information Sheet and Consent forms shared. The 40–60min interview was carried out in the Conference Room of BMH by the principal researcher after obtaining consent from the participants and the each FGD.The recorded discussion was transcribed into text in Microsoft Word after the interview was completed. The raw data was entered in the Word document. The remaining relevant data was analyzed using thematic analysis whereby the text was grouped into common theme related to the topic (Farmer et al., 2015). Using thematic content analyses, the transcripts were read several times to be familiar with the data and a set of common themes were developed to describe groups of categories with similar meanings (Dougherty, 2018; Farmer et al., 2015).

Ethical approval (ID: 199.18) was obtained from the College of Health Research Ethics Committee (CHREC) of Fiji National University and the Ministry of Health's Fiji National Heath Research Ethics Review Committee (FNHRERC).

## 3 Results

### 3.1 Male partners

There were a total of 10 male participants from the 2 FGDs. The mean age of male participants was 26.8 years (SD = ± 7.32) with minimum age of 19 years and maximum 41 years. Majority of them were in the 20–24 years old (30%) age group. Majority of the men were of Hindu faith (40%) primarily Fijians of Indian descent (60 %). 70% of the employed men were from urban area. 50 % men had secondary level education. Most of the men (70%) were married and 40% men fell in the 1–4 years of marriage (Table 1).

**Table 1:** General characteristics of male partners.

Characteristics	Categories	Frequency	Percentage
Age (Years)	18–19	2	20
	20–24	3	30
	25–29	2	20
	30–34	1	10
	35–39	1	10
	40–44	1	10
Religion	Christianity	3	30
	Hindu	4	40
	Islam	3	30
Ethnicity	iTaukei	4	40
	Fijian of Indian descent	6	60
Employment	Employed	7	70
	Unemployed	3	30
Residency	Rural	3	30
	Urban	7	70
Education	Primary	3	30
	Secondary	5	50
	Tertiary or higher	2	20
Marital status	Married	7	70
	Defacto	3	30
Years of Marriage	<1	1	10
	1–4	4	40
	4–7	3	30
	7–10	1	10
	>10	1	10

The views of the participants towards contributing factor of FP were classified under themes.

***Theme 1: Opposition of Male Partners***

The male partners were asked whether they support or oppose their wife in FP and one of the participants mentioned about the effect of cultural and family beliefs against FP use*.*

*“It is against my belief for women to use contraception, as in our family, the females never used contraception”* (31 year old, Fijian of Indian descent).

Further, the participant was asked as to how he knew that in his family no one used contraception and he shared his father experience with his mother.

*“I had a chat with my dad when I got married and he said that my mum never used it and that my dad never allowed her to use due to side effects as he said if women take contraception, they will get fat”* (31 year old, Fijian of Indian descent).

Another participant said about the ratio of boys to girls in the family as a reason for not supporting his wife to take contraception. He mentioned that:

*“I want boys in family and so did not want the wife to take contraception until we had a boy. My wife got pregnant 8 times and all were girls and then the ninth baby was a boy so then we had 2 more boy after that’’* (41 year old, iTaukei male).

One of the participant said that side effects was the reason he did not favour his wife to use contraception:

*“I have heard that there are a lot of side effects especially weight gain and so I do not want my wife to get fat and so due to side effects, I do not encourage my wife”* (24 year old, Fijian of Indian descent).

However, only some said that they do support their wife with contraception as one of them said:

*“I support my wife with family planning as I believe we need to help our wives in this and I also take her to clinics and ask about which contraception will you take and so forth”* (21 year old, Fijian of Indian descent).

***Theme 2: Religious norms and cultural beliefs***

The men were asked about their view on religious norms and cultural beliefs towards contraception and one of the participant stated that it was not in their religion to use FP and that has been practiced over the generations:

*“In my religion, it is not allowed, my mum never used it and so I told my partner not to use as it is forbidden in our culture”* (19 year old, iTaukei Male).

While one of them said the opposite of the previous one as there are no objections in the religion:

*“In my religion, there are no objections to FP as there is nothing mentioned in the holy books that contraception is Haram”* (36 year old, Fijian of Indian descent).

One of the participants said that religious belief does have an effect on the use of FP as he mentions that:

*“In our religion, the more the children, the better as Prophet said that the more the children there is, the more the believers will grow”* (24 year old, Fijian of Indian descent)

***Theme 3: Accessibility of contraception***

Male partners were asked about their views on accessibility of contraception and one partner mentioned that availability is not as issue as it is available in public and private facilities. He shared that

*“I get it from health centres or buy it. I know it's available at both the places and which is available to me I get it from there”* (27 year old, Indo-Fijian male).

And one of them said that he does not access it usually and prefers the wife to get it:

*“I ask the wife to get it for them as I am busy at work so I ask her when that when she goes to clinics, to get condoms for us”* (31 year old, iTaukei Male).

While one said that he was ashamed to go and ask or buy it and so doesn't get it by himself:

*“I am ashamed to go and ask for it so I don't get the contraception”* (21 year old, Fijian of Indian descent).

He was further asked as to why he was ashamed as it is a medical facility and he mentioned that:

*“I am ashamed as there are usually female staff and I feel shy to go and ask them and there are hardly any male staff in the contraception section”* (21 year old, Fijian of Indian descent).

And one said about the availability of the contraception as usually it's not available in the rural health facilities:

*“It's not in the health centre in my village and so I cannot go to town and buy just that as my village is far from town and so most of the time I just don't use since not available”* (24 year old, iTaukei).

Hence, it shows that some do take the initiatives to access it while others do not access.

***Theme 4: Previous FP failure***

For previous FP failures, the male partners were asked about what they think as to why FP is not practiced and one of them said that because of the unavailability in the remote areas he doesn't use it:

*“I stay in rural area and it's not readily available, that is why i don't practice it as it’s not available at time of need”* (36 year old, iTaukei male).

And another participate mentions about the side effects experienced as a reason of not using it anymore:

*“…due to side effects like weight gain, headaches, I do not want my wife to practice it*” (27 year old, Indo-Fijian male).

Another participant mentioned that to have a male child was the reason why he did not want the wife to use any FP methods:

*“the desire to have a boy, keeps me having more kids as I want to have more than 3 kids so we don't use FP”* (41 year old, iTaukei male).

Lastly one said that the fear of the wife getting pregnant as despite using contraception, the wife got pregnant:

*“my wife and I used it and it failed and so we don't want to use it”* (27 year old, I-Taukei male).

### 3.2 Medical staff

There were a total of 10 participants from the 2 FGDs. The mean age of the respondents were 30.4 years (SD = ± 4.77) with minimum age of 25 years and maximum 41 years. Majority of the respondents were in the 25–29 years old (50%) age group. Majority of the medical staff were of Hindu faith (40%) and most were Fijian of Indian descent (70%). 40% of medical staff fell in the 1–4 years of work experience (see Table 2).

**Table 2:** General characteristics of medical staff.

Characteristics	Categories	Frequency	Percentage
Age (years)	25–29	5	50
	30–34	3	30
	35–39	1	10
	40–44	1	10
Religion	Christianity	3	30
	Hindu	4	40
	Islam	3	30
Ethnicit	iTaukei	3	30
	Fijian of Indian Descent	7	70
Years of Experience	<1	1	10
	1–4	4	40
	4–7	3	30
	7–10	1	10
	>10	1	10
Post	Doctor	2	20
	Midwife	5	50
	Staff Nurse	3	30

The views and perceptions of the participants towards contributing factors of FP were classified under themes.

***Theme 1: Effectiveness of Counselling***

To determine the effectiveness of counselling, the participants were asked their views of FP counselling that was rendered for reproductive age group and one of the participants responded about the counselling services at antenatal and postnatal:

*“I have been doing the counselling at ANC every week and I think it is effective, but most of the mum are not counselled in post-natal after discharge and at follow up by the nurses”* (26 year old, Medical Officer).

While one of the participants said about time being a major factor in counselling for the women in the clinics:

*“I did counselling few times in the clinic and due to the large number of mothers who come for clinics, usually I could not spend that much time with them and so I think we need to spend some more quality time with them to have a better counselling session with them”* (29 year old, Staff Nurse).

One of the Staff Nurse shed light on importance of couple counselling:

*“I am of the view that we need to counsel the couple together once during the clinic so that we can inform the partners of the importance of FP, hence there can be a better understanding between the couple as currently we are just counselling the women mostly”* (31 year old, Medical Officer).

Furthermore, when asked about how they are followed up after delivery, it was said by one of the participants that about the need for FP specialised nurses:

*“the staff nurses are not specialised for counselling and hence there needs to have specialised FP nurse for counselling and all the patients after discharge need to be followed up and counselled properly”* (26 year old, Staff Nurse).

***Theme 2: Concerns on men involvement in FP***

To understand the concerns relating to men involvement in FP, the staff was asked about what they think about men's involvement in FP as they highlighted that men play an important part in the decision making for FP and one of the staff responded that:

*“I think the men should be involved in FP counselling and lectures in ANC as it is important for them to know what is FP and what choices are available and hence they could make a decision together”* (30 year old, Medical Officer).

Another staff mentioned about the need for men to be more involved in the clinics and their participation:

*“In my opinion the men should be encouraged to accompany the women during booking so that they know what the women decide and they should also sign with the women on the preferred method for contraception”* (35 year old, Staff Nurse).

When asked to elaborate, she said male partners have limited involvement in FP decision making and highlighted the phase when they are involved the most:

*“…umm...Men are only asked to counter for Tubal Ligation and so it is important that male partners also sign on for contraception at booking after delivery with women”* (35 year old, Staff Nurse).

One of the Medical officer highlighted that about the role of men in FP post-delivery as:

*“I think Men need to be counselled together with women at post-natal and also in Post-natal clinic so they take responsibility of FP towards women also”* (35 year old, medical officer).

***Theme 3: Barriers towards FP***

To determine the barriers towards FP, the medical staff was asked about what they thought of the barriers that affect FP and one of the staff said that cultural and traditional beliefs are one of the reasons of not using FP:

*“I think some couples have traditional and cultural beliefs that act as a stigma for them and expect them not to practice FP, and in their beliefs practicing FP is a taboo and should not be practiced”* (29 year old, Staff Nurse).

And another participant said access of the contraception plays an important part:

*“Some women are not able to access contraception readily due to transportation or no health facilities available near their place and hence if they do not have any contraception available then they do not use it”* (35 year old, Midwife).

Another staff mentioned some women have the fear of getting pregnant despite using FP:

*“Some women do not practice FP due to previous failures or side effects as they got pregnant even though they used it so the fear of them suffering the same fate, so they do not use it.”* (31 year old, Medical Officer).

Furthermore, one of the respondents mentioned about the role of male partners in FP and that disapproval from them was another factor:

*“Some women also do not use FP because their husbands do not approve of it and even though they know what contraception are available, they cannot use it as their husbands disapprove it”* (26 year old, Staff Nurse).

One of the staff mentioned that attitude of the medical staff is also a barrier of FP use:

*“Some women do not practice or are not comfortable due to the attitude of the medical staff as sometimes the staff are unfriendly or unapproachable so they do not make an effort to seek FP”* (25 year old, Staff Nurse).

When asked to elaborate on this, she added that poor attitude towards patients is one of main reasons patients do not seek advice:

*“The patient was sharing with me and she said that whenever I go to the clinic and ask about FP to the nurse, she would get angry and not tell me much and so I did not ask her again about FP”* (25 year old, Staff Nurse).

## 4 Discussion

The qualitative results show the perception of the male partners and the medical staff in regards to FP. It showed that male partners had a mixed perception of FP. While some of them were in favour of FP, others did not approve of FP. Undeniably, male partners play a crucial role in FP as they are involved in decision making with the women and their perception on FP would be determined on the knowledge of FP among men. Lack of knowledge about FP highlighted in this study was noted in a study by Dougherty (2018) in order to determine the knowledge of male partners on myriad contraceptions and its side effects and it was noted that most common method known by men was the male condom (72%), but more than half also knew of injections (54%) and pills (52%). Kaitani (2012) study of men involvement in FP in Fiji shows quite convincingly that men's knowledge FP is much poorer than women's and that they tend to have much more negative attitudes towards FP. Adelekan et al. (2014) also showed a similar finding.

Moreover, the negative attitude highlighted in this study towards FP was seen in another study by Kabagenyi et al. (2014) noted that five reasons related to men's attitude that lead to limited involvement in FP and these included; perceived side effects of female contraceptive methods which disrupt sexual activity, limited choices of available male contraceptives, including fear and concerns relating to vasectomy, perceptions that reproductive health was a woman's domain due to gender norms and traditional FP communication geared towards women, preference for large family sizes which are uninhibited by prolonged birth spacing; and concerns that women's use of contraceptives will lead to extramarital sexual relations. Kaur et al., (2015) and Shih et al., (2011) showed a similar finding.

Mboane and Bhatta (2015) reported that women who said that their male partners made the decision about FP were 19% less likely to use contraception as compared to women who had joint decision making. Likewise, Durowade et al. (2017) reported that 25.5% women highlighted that they can't practice FP as male partner disapproves. Yet in another study by Gonie (2018) found that barriers identified with the low contraception rates were opposition from spouse (38.3%). Also Apanga and Adam, (2015) found that 90% of women reported that the major reason for not utilising FP services was due to opposition by male partner as they did not approve for wife to use FP as some wanted more children and some disapproved due to cultural beliefs. In addition to this, Tilahun et al. (2013) highlighted that 72% of men wanted more children compared to 64% women. Hence, it shows that men do have a strong influence on women decision towards FP and women still feel that they are not able to make decision on their own.

Furthermore, this disapproval of FP usage by men found in this study was also noted in a similar study done by Lincoln et al. (2018) among women in Suva, Fiji, it was noted that 20.3% of the women from the present study reported that their male partners object to the use of contraceptives while 25.2% find it embarrassing to talk about contraception with their partners. In other words, a fifth to a quarter of women in Suva, Fiji finds the largest obstacle for the use of contraceptives in their own male partners, even those women who do not face this challenge themselves. Also, women who do not report that their male partners object to their use of contraceptives are still aware that many other women do face such an obstacle. As much as 89.5% of women believe that a change in male attitudes would lead to an improvement in the use of contraceptives, and 83% of them believe that male partner's objections sometimes prevent women from using contraceptives.

The men are not always against FP and there are men who do support FP and are always for the use of FP and sometimes take the lead in making decision for the FP. The current study noted that some men were in favour of FP use and do support women with their decision of FP use. A similar finding was noted in a study by Mattebo et al. (2016) that some men chose to go against their family, culture and their own interests in order to support women. A similar finding was noted by Kofi et al., (2018) as it was highlighted than men were strongly supportive of FP due to socioeconomic issues and believe that the decision should not be unilateral. This study shows that the perception of men towards FP was positive.

The finding from these studies are similar to the finding of the current study as males have a mixed perception as some are opposed to FP due to cultural beliefs, fear for side effects, had bad past experiences and failures, some have preference for gender and want more kids and believe it's only for women. However, there was support also noted for FP among men as they believed that FP should be a joint decision making concept and that females should not only be responsible for it.

A qualitative study by Kofi et al. (2018) showed that limited method choice for men, insufficient venues to receive services and few messages that target men create barriers for male engagement in family planning. This shows that men are not aware of the choices they have available for them and so they are not motivated in the FP practice for themselves. The author goes on to mention that men do need more awareness and education on FP.

The medical personnel play an important role, since they are directly involved with counselling and creating awareness on the use of contraception among women whether in the clinic, Maternity Wards, or during Outreach Programmes. The public relies on medical personnel to provide FP service as they have the required knowledge and skills to provide the service and hence the men and women of the reproductive age group seek help from them (Japaridze, 2015).

This study depicts that medical staff were well versed with the types of contraception available, hence providing preference of contraception they viewed best suited for women. This demonstrates the staff knowledge and that should not be a factor for hindrance in FP service. Htay et al. (2018) showed that in a study among final year medical students believed that the reason for poor practice for FP is due to cultural barrier, misconception, inadequate knowledge, and improvement for the health-care services.

Moreover, the importance of men in FP has been discussed in a study by Shih et al., (2011), it was noted that men were hardly involved in the FP counselling and that the option of vasectomy is hardly discussed during the counselling of FP with women. This study showed that medical staff impressed upon the need for male partners to be involved in the counselling from the clinic and continue this even after the delivery of the wife so that they have a responsibility towards their wife/partners. Indisputably, men would benefit from joint counselling as highlighted by the staff.

The medical staff in this study highlight that counselling services need to be strengthened with emphasis on its significance in order to ascertain a desired outcome. It was also highlighted that the staff are not trained in counselling and that needs to be taken into consideration for them to have a better counselling session and also if a full time FP Is available then would be to provide a better counselling with the time and skill available. It was also highlighted that the FP counselling in the postnatal phase needs to be improved and that men need to be involved in the counselling sessions for a better outcome. This suggestion is also agreed by men as discussed in the previous section. A similar finding is from this study as men believe they need to be more involved in FP counselling, need more interaction with the medical staff, they need to be involved in counselling post-delivery and they need more awareness on FP (Harrington et al., 2016; Ayiasi et al., 2015 and Shih et al., 2011).

The participants from the study highlighted that in order to improve the service delivery there needs to be an improvement in counselling services provided to both women and men. Secondly there needs to be a full time FP nurse who could be committed to both prenatal and postnatal counselling and have a better follow up of cases. Moreover, there needs to be more training sessions for the staff to help develop professionally. Also, there needs to be a better availability of resources to assist women and men to have options available for contraception and it is more accessible. Finally, males needs to be engaged and cognizant of the postnatal clinic and after delivery in terms of counselling and for them to become more responsible towards FP (Htay et al., 2018; Sharma et al., 2012; Silumbwe et al., 2018).

### 4.1 Limitations

This study focused on male partners’ perception about contributing factors of FP so that the participants might find it difficult to share all the factors due to cultural or personal barriers in the FGD. . Another challenge was the sample size. It was a small group due to the less number of staff at BMH and the unavailability of partners of the pregnant women during clinical days.

## 5 Conclusion

From this study it was noted that men have a mixed perception towards FP and that some still have negative perception when it comes to FP practice due to barriers like cultural and religious beliefs, accessibility and past experiences. However, the perception of medical staff is that there is need for a strengthening of counselling and more involvement of men. These can be further explored in future studies. The findings from the study can be used to improve the FP uptake in Fiji, involvement of men in FP during antenatal and postnatal, and create more training for medical staff in FP in the medical facilities.

## Declarations

### Author contribution statement

M. Imtishal: Conceived and designed the experiments; Performed the experiments; Analyzed and interpreted the data; Contributed reagents, materials, analysis tools or data; Wrote the paper.

M. Mohammadnezhad: Conceived and designed the experiments; Analyzed and interpreted the data; Contributed reagents, materials, analysis tools or data; Wrote the paper.

### Funding statement

This research did not receive any specific grant from funding agencies in the public, commercial, or not-for-profit sectors.

### Data availability statement

Data will be made available on request.

### Declaration of interests statement

The authors declare no conflict of interest.

### Additional information

No additional information is available for this paper.
